# Linear doggybone DNA vaccine induces similar immunological responses to conventional plasmid DNA independently of immune recognition by TLR9 in a pre-clinical model

**DOI:** 10.1007/s00262-017-2111-y

**Published:** 2018-01-12

**Authors:** Alex Allen, Chuan Wang, Lisa J. Caproni, Gessa Sugiyarto, Elena Harden, Leon R. Douglas, Patrick J. Duriez, Kinga Karbowniczek, Jon Extance, Paul J. Rothwell, Ifeayinwa Orefo, John P. Tite, Freda K. Stevenson, Christian H. Ottensmeier, Natalia Savelyeva

**Affiliations:** 1 Cancer Sciences Unit and Cancer Research UK and Experimental Cancer Medicine Centre Protein Core Facility, Faculty of Medicine, University of Southampton, Tremona road, Southampton, SO16 6YD UK; 2 Touchlight Genetics Ltd, Morelands and Riverdale Buildings, Lower Sunbury Road, Hampton, London, TW12 2ER UK

**Keywords:** Doggybone DNA vaccine, Bacteria free production, Cancer vaccine, CD8+ T cells, TLR9, STING

## Abstract

Vaccination with DNA that encodes cancer antigens is a simple and convenient way to raise immunity against cancer and has already shown promise in the clinical setting. Conventional plasmid DNA is commonly used which together with the encoded antigen also includes bacterial immunostimulatory CpG motifs to target the DNA sensor Toll-like receptor 9. Recently DNA vaccines using doggybone DNA (dbDNA™), have been developed without the use of bacteria. The cell-free process relies on the use of Phi29 DNA polymerase to amplify the template followed by protelomerase TelN to complete individual closed linear DNA. The resulting DNA contains the required antigenic sequence, a promoter and a poly A tail but lacks bacterial sequences such as an antibiotic resistance gene, prompting the question of immunogenicity. Here we compared the ability of doggybone DNA vaccine with plasmid DNA vaccine to induce adaptive immunity using clinically relevant oncotargets E6 and E7 from HPV. We demonstrate that despite the inability to trigger TLR9, doggybone DNA was able to induce similar levels of cellular and humoral immunity as plasmid DNA, with suppression of established TC-1 tumours.

**Electronic supplementary material:**

The online version of this article (10.1007/s00262-017-2111-y) contains supplementary material, which is available to authorized users.

## Introduction

DNA vaccines are emerging as a promising vaccine modality for cancer. The DNA vaccine concept is simple: a bacterial plasmid DNA is used which contains the target antigen under the control of a strong eukaryotic promoter enabling expression in eukaryotic cells. Following injection of the DNA vaccine, the encoded antigen is expressed directly in the muscle or skin without the need to employ other expression systems [1]. The second critical component of DNA vaccines is the ability to stimulate innate immunity through recognition of the DNA itself. Multiple sensors that recognise DNA exist in the cytoplasm in addition to the endosomal DNA sensor TLR9 [2, 3]. While many types of DNA, mainly derived from viral sequences, can stimulate cytoplasmic DNA sensors, TLR9 recognises specific immunostimulatory sequences (ISS); hypomethylated CpG motifs [3, 4]. These are frequently found in bacterial DNA but not commonly in vertebrate DNA and are often located within antibiotic resistance genes. The result of stimulation through these sensors is induction of inflammatory cytokines and type I interferons which are responsible for the self-adjuvanting effect of DNA vaccines [5]. Despite the relative simplicity of DNA vaccines they are able to induce both the durable humoral immunity and cytotoxic T cells desired for cancer attack [1].

Although DNA vaccines performed well in mice, the conditions of injection in patients differed and led to limited immunogenicity. Several approaches to improve immunogenicity of DNA vaccines have been developed [6, 7]. An important strategy was to combine injection with in vivo electroporation (EP) which significantly improved immunogenicity by increasing cell transfection, leading to improved antigen expression and adjuvancy [7, 8]. Robust immunological responses after DNA vaccine plus EP have been demonstrated in patients, with indication of clinical efficacy [9, 10]. A recent phase IIb clinical trial using a DNA vaccine encoding HPV oncogenes E6 and E7 combined with EP has demonstrated regression of dysplastic processes in patients with HPV-driven cervical dysplasia [11].

Traditionally DNA vaccines relied on plasmid (PL) DNA prepared using *Escherichia coli* with an antibiotic resistance gene for selection. A subsequent multiple step purification is required followed by endotoxin removal if intended for clinical use. Recently a bacteria-free manufacturing platform has been developed to allow rapid production of novel doggybone™ DNA (dbDNA™), which is suitable for use as a DNA vaccine. The method involves an enzymatic amplification in vitro using two enzymes. Phi29 DNA polymerase is employed to rapidly amplify template DNA into concatamers and then the protelomerase TelN from bacteriophage N15 is used to cut and join the DNA concatamers into individual closed linear dbDNA™ [12, 13]. The resulting DNA is fully functional, highly stable and contains only the minimal sequences required including the antigenic sequence, a promoter and a poly A tail but lacks bacterial sequences such as the antibiotic resistance gene. Although this is advantageous for patients’ safety the question of immunogenicity arises since the innate immune recognition could be compromised due to decreased ISS frequency. This is especially relevant for cancer antigens delivered through DNA vaccines as these are of non-bacterial origin and hence often lack ISS.

In this study, we compared the immunogenicity of a dbDNA™ vaccine (DB) targeting HPV16 derived E6 and E7 oncogenes to conventional PL delivery and look into the potential pathways involved in innate sensing of this novel DNA vaccine.

## Methods

### Preparation of DB and PL DNA vaccines

The HPV16 E6 and E7 sequences containing mutations that impair oncogenic potential were assembled as previously [14] and the E6E7 fusion was cloned into the proTLx™ based PL. The proTLx™ PL consisted of the CMV promoter plus enhancer, a multiple cloning site and an SV40 late polyadenylation signal flanked by 2 telRL sequences, the site of protelomerase TelN recognition and cleavage. The PL backbone contained an ampicillin resistance gene and the pUC  origin of replication. The resulting template PL was verified by sequencing and maintained in recombinase-deficient *E. coli*.

The PL was purified using an endo-free maxiprep kit (Qiagen). The template plasmid was denatured using 0.1M NaOH for 5 min at 30 °C and then quenched in reaction buffer (30 mM Tris–HCl pH 7.4, 5 mM (NH_4_)_2_SO_4_, 30 mM KCl, 7.5 mM MgCl_2_, 2 mM dithiothreitol) containing 50 µM custom primer (Metabion), 4 mM dNTPs (Bioline), 200 units/ml Phi29 polymerase (Enzymatics) and 0.05 units/ml pyrophosphatase (Enzymatics). Upon mixing, the reaction was incubated at 30 °C for 30 h. Concatameric DNA was processed by addition of 2 mM TelN protelomerase (Enzymatics). Further processing was performed using 200units/ml *Xba*I (Enzymatics) and 200units/ml exonuclease *Exo*III (Enzymatics). The digest mixture was cleaned from reaction components by addition of 500 mM NaCl/100 mM MgCl_2_ and precipitated using 3.9% polyethylene glycol (PEG) 8000 (Applichem). DNA was pelleted (10 min, 4500 g) and re-suspended in 20 ml 500 mM NaCl/100 mM MgCl_2_. The 3.9% PEG 8000 precipitation was repeated with final resuspension in water prior to ethanol precipitation to remove residual salts.

### Vaccination protocols

C57Bl/6 mice were used in accordance with U.K. Home Office Guidelines, following the Animals (Scientific Procedures) Act 1986. Ethical approval was obtained from the Science Review Group in Southampton and the Animal Welfare and Ethical Review Board. At each prime or boost vaccination, the mice were vaccinated in both hind legs. DNA was diluted in 100 µl saline (50 µl containing 25 µg of DNA per injection). I.m. injections without EP were performed in the anterior tibialis and injections with EP were given in the quadriceps muscle. EP was carried out on mice anaesthetised by isofluorane, using a custom-made pulse generator from Inovio Pharmaceuticals. 10 trains of 1000 square wave pulses were delivered at a frequency of 1000 Hz, each lasting 400 µs (200 μs positive and 200 μs negative). Each train was delivered at 1 s intervals, with the electrical pulse kept constant at ± 50 mA. Booster injections were given 7 weeks after priming as this time point was optimal for generating T-cell responses with DNA vaccines (unpublished data).

### Tetramer staining

50–100 µl blood was taken into a heparin containing buffer (100 U/ml Heparin, 10 mM EDTA, in PBS). Red blood cells were lysed using RBC lysis Buffer (Qiagen). After incubation with anti-CD16/32 blocking antibody (clone 93, eBioscience) PE-labelled H2-D^b^-E7_49–57_ tetramer made in house was added 15 min before an allophycocyanin (APC)-labelled anti-CD8a antibody (clone 53 − 6.7, Biolegend). Samples were run on a FACS Canto I (BD Bioscience).

### Tumour suppression and T-cell subset depletion

The TC-1 mouse lung epithelial cell line was cultured in complete RPMI 1640 media (10% FCS, 4.5 g/l glucose, 2 mM glutamine, 50 U/ml penicillin and 50 µg/ml streptomycin). TC-1 cells express E6 and E7 and are co-transformed with Ras [15].

C57Bl/6 mice aged 10–13 weeks were injected s.c. with 5 × 10^4^ TC-1 cells in 200 µl of PBS, on d 0. Three days later the mice received 50 µg E6E7 DB or PL DNA, or an irrelevant enhanced GFP (eGFP)-encoding DB, with or without EP. Naive mice were included as controls. Tumour measurements were taken every 3–4 days, and mice culled when tumours reached a mean diameter > 15 mm.

In T-cell subset depletion experiments one dose of DB vaccine was given with EP 4 weeks prior to TC-1 tumour challenge (as above). The depleting CD8 antibody (YTS169.4) or isotype control (both 700 µg per mouse, from BioXcell, West Lebanon, USA) was given i.p. 1 day before and 6 days after tumour challenge.

### ELISPOT

Induction of peptide-specific CD8+ or CD4+ T cells in individual mice was assessed ex vivo using an IFN-γ ELISPOT kit or IL-4 ELISPOT kit (CD4+) (both from BD Pharmingen). Briefly, splenocytes (2.5 × 10^5^ cells/well) were incubated in complete RPMI for 22 h with specific or control peptide, or peptide library (1 µM for each peptide). Overlapping peptide pools covering the E6 and E7 sequence consisted of 15-mers with an 11 amino acid overlap at > 95% purity (GL Biochem; sequences in Supplementary table 1). Control wells were incubated without peptide to assess background. Samples were plated in triplicate and mean values were expressed as spot-forming cells (SFCs) per 10^6^ cells. Levels were considered positive if at least two times above background.

### ELISA for IgG and IgG isotypes

Serum samples were collected 2 weeks after boosting. ELISAs were performed as described elsewhere [16]. Briefly, Maxisorp Immuno plates (Nunc) were coated with 3 µg/ml E6 or E7 protein overnight at 4 °C. Both proteins were expressed in house in *E.coli*. For E6 the pET41M-HPV16-E6_C4S expression vector was used (kind gift from Dr G. Travé, Ecole Supérieure de Biotechnologie, Bd Sébastien Brant, France) and the protein purified following the published protocol [17]. Expression vector pGEX2T-E7 [18] (Addgene plasmid # 13634) was kindly provided by Dr K. Munger (Tufts University, Boston, USA). E7 protein was purified using glutathione beads (GE Healthcare) following the manufacturer’s protocol. Serum antibody bound to the coated antigen was detected with HRP-conjugated anti-mouse IgG (The Binding Site), anti-IgG1 (Oxford Biotechnology), anti-IgG2b or anti-IgG2c (both from Harlan Sera-Lab) as previously [16, 19].

### Assessment of innate immune recognition of the DB DNA vaccine

HEK-Blue cells expressing human or mouse TLR9 (InvivoGen) were cultured in complete DMEM supplemented with 100 µg/ml Normocin, 30 µg/ml Blasticidin and 100 µg/ml Zeocin (all InvivoGen). HEK-Blue cells contain a secreted embryonic alkaline phosphatase (SEAP) reporter system, which generates a colorimetric change following TLR9 engagement.

Transfections were performed with Fugene (Promega), following the manufacturer’s protocol. Cells were plated at 6 × 10^4^ cells per well in a 96-well plate in HEK-Blue detection media (InvivoGen) or complete DMEM. 300 ng of E6E7 DB or PL DNA complexed with Fugene (5 µl/well) was added directly to the cells. In selected wells human A151 (InvivoGen) or mouse 4084-F (InvivoGen) TLR9 antagonists were used at 10 µg/ml. After 42 h A_625nm_ was measured. To confirm transfection efficiency was similar between DB and PL vaccines eGFP encoding constructs were used, with eGFP expression assessed using a fluorescent microscope.

To investigate the involvement of cytosolic DNA sensing pathways, the THP1-Blue ISG reporter cell line and the THP1-Blue ISG-KD-STING cell line which has STimulator of INterferon Genes (STING) knockdown, were used (both InvivoGen). Transfections were performed as above and 24 h later the cell supernatants  were incubated with QUANTI-Blue reagent for 25 min and A_625nm_ was measured.

### Bone marrow derived dendritic cell (BMDC) generation and activation

DCs were prepared from bone marrow BM collected from the tibias and femurs of 6–8 week old mice. BM from MyD88 knock out (KO) mice was kindly provided by Dr S. Nanda (University of Dundee, UK) with the permission of Prof S. Akira (University of Osaka, Japan). BM from STINGKO mice was kindly provided by Dr J. Rehwinkel (University of Oxford, UK) with the permission of Prof J. Cambier (University of Colorado, USA). DCs were generated using the CellXVivo DC differentiation kit (R&D). Briefly, BM cells were plated at 10^6^ cells/ml in media containing 800 IU/ml GM-CSF and 500 IU/ml IL-4, and cultured in 6-well plates for 6 days with the media changed every 2 days before using in activation experiments.

BMDCs were treated with 1 µg/ml of DB or PL DNA with 1 µg/ml lipofectamine 2000 (Thermofisher) or left untreated, as in [20]. Transfection efficiency was checked by FACS using DB or PL DNA encoding eGFP. LPS and poly(I:C) were used as controls at a final concentration of 1 µg/ml. After 20 h supernatants were collected for ELISA or Luminex. BMDC collected by centrifugation were used for FACS to check upregulation of CD40, CD80 and CD86, and for RNA purification.

### Real-time PCR (qPCR)

To evaluate gene expression RNA from 5 × 10^6^ purified DC was extracted using the RNeasy kit (QIAGEN). RNA was reverse transcribed to cDNA using a high capacity cDNA reverse transcription kit (Thermofisher). mRNA levels of IL-12a, IL-12b, IL-10 and C-X-C motif chemokine ligand (CXCL)-10 [IFN-γ-inducible protein 10 (IP10)] were assessed by real-time PCR following the protocol for TaqMan™ Fast Advanced Master Mix (Thermofisher) and performed using an Applied Biosystems 7500 Real-Time PCR system (Thermofisher). All qPCR 6-FAM-labelled (6-carboxyfluorescein) primers were purchased from Thermofisher.

### Evaluation of type I interferons in DC experiments

IFN-α in BMDC supernatants was measured using a Luminex bead assay kit (Life Technologies) using a Bio-Plex^®^200 system (Bio-RAD). IFN-β levels were assessed using the LEGEND MAX™ Mouse IFN-β ELISA Kit (Biolegend). Samples were diluted in assay diluent as required. Each sample was assayed in triplicate and cytokine standards supplied by the manufacturer were used to calculate the concentrations of the samples.

### Statistics

The Mann–Whitney statistical test was used for T-cell responses and the antibody data, and the Mantel–Cox test was used to compare survival. For in vitro data the unpaired *t* test was used.

## Results

### Induction of CD8+ and CD4+ T-cell responses by the DB DNA vaccine

**Fig. 1:**
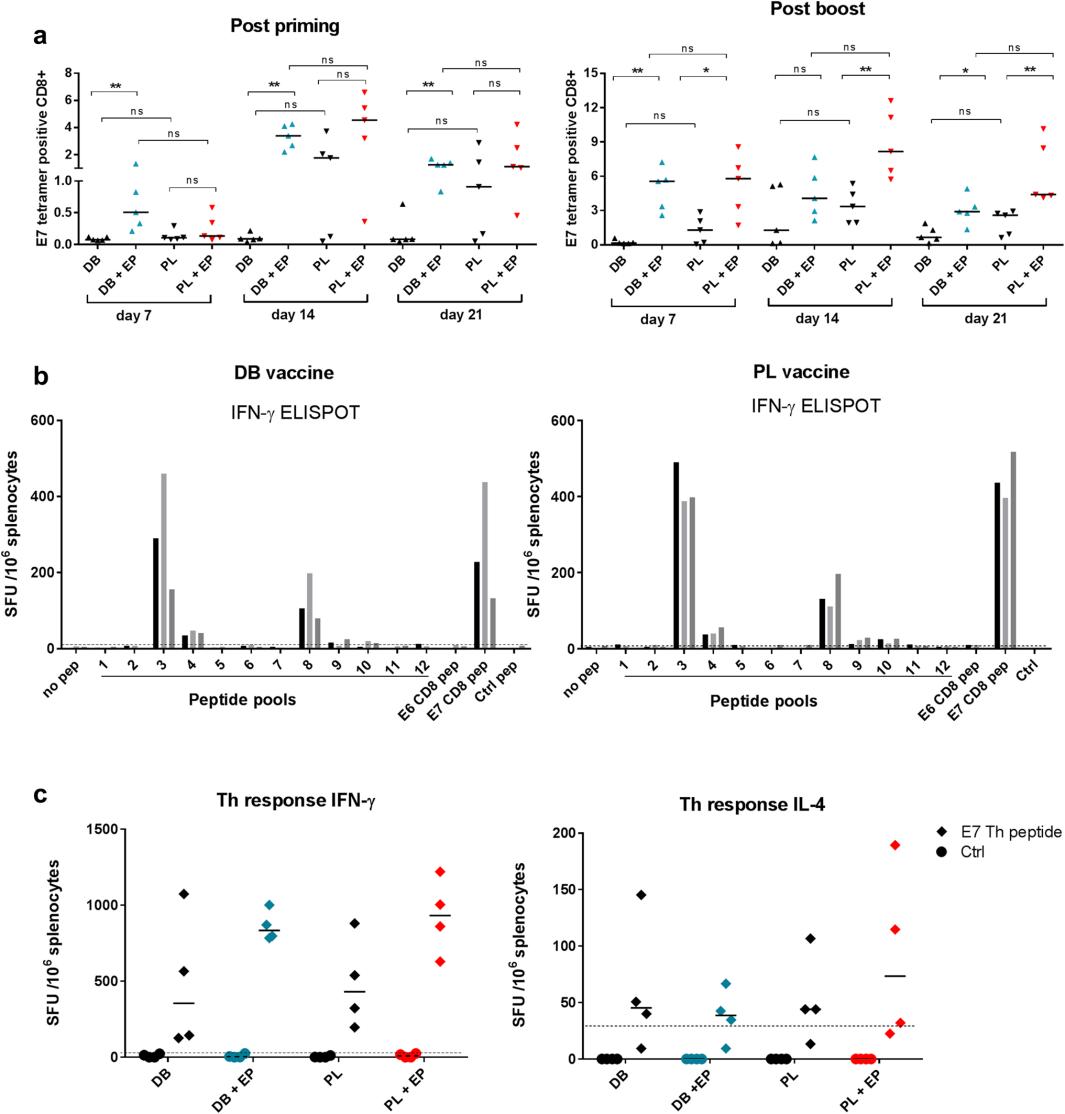
Evaluation of CD8+ and CD4+ T cell responses after vaccination with DB or PL DNA vaccines encoding E6E7. Mice were injected i.m. with 50 µg of the E6E7 DB or PL vaccines with or without electroporation. **a** Serial blood samples were analysed using an E7_49–57_ tetramer after priming (left panel) and after boosting (right panel). Values are tetramer positive cells as a percentage of total CD8+ cells. The results are representative of 2 independent experiments each with *n* = 5 mice per group. *p < 0.05, **p < 0.01, *ns* non-significant. **b** Evaluation of CD8 + responses by IFN-γ ELISPOT after priming and boosting using overlapping peptide pools for E6 and E7. E6 pep and E7 pep are the immunodominant peptides E6_48–57_ and E7_49–57_, respectively. Graph is representative of 2 independent experiments each with *n* = 3 mice per group. Bars represent individual mice. **c** CD4+ Th responses were measured after priming and boosting using IFN-γ or IL-4 ELISPOT using the E7_43–77_ peptide containing a well characterised Th epitope E7_44–60_. Plotted values have the non-specific background (media alone) subtracted. The cut-off line represents two times the response from naive control mice

To evaluate the induction of CD8 responses by the DB DNA vaccine we used DNA that encodes E6E7 fusion from HPV16. This vaccine includes the H-2D^b^-binding E7_49–57_ epitope RAHYNIVTF [21] and hence we employed PE-labelled H2-D^b^-E7_49–57_ tetramer staining to evaluate CD8 responses. Mice were injected with 50 µg DB DNA alone or DB DNA followed by EP. For comparison a conventional PL DNA vaccine encoding the same E6E7 fusion was used with or without EP. Mice were bled weekly at time points indicated in Fig. 1a. Without EP both DB and PL performed poorly with PL inducing higher levels than DB (Fig. 1a, representative tetramer staining Supplementary Fig. 1). There was more impact of EP on DB, already demonstrating a significant improvement at day 7 post priming when PL did not yet show significant responses. Post priming both DB and PL with EP peaked at day 14 while DB without EP never rose above baseline. PL without EP also peaked at day 14. EP was required for DB to induce CD8, while PL showed less dependency on EP. Post boost responses were significantly enhanced by EP for PL and DB, with a more pronounced boosting effect in comparison without EP. Overall, DB and PL induced similar levels of specific CD8 T cells and this was true with or without EP, with a trend of lower responses produced by DB without EP.

To evaluate whether E7_49–57_ was immunodominant in this setting we used an overlapping peptide library for E6E7 where the immunodominant E7 epitope was part of pool 3 (Supplementary table 1). The responses were evaluated by IFN-γ ELISPOT (Fig. 1b). Clearly the majority of reactivities were induced to pool 3 in all mice tested, with similar levels as to the E7 peptide. A lower response was induced to pool 8 which contained the E6 immunodominant epitope (E6_48–57_) [22]. No reactivities were observed to another well-characterised E6 epitope YRDGNPYAV which was in pool 9. Both DB and PL demonstrated a similar pattern of reactivities with the majority of responses to the E7 immunodominant epitope.

CD4 Th responses were evaluated by ELISPOT as previously described using a long peptide containing the well-defined T-helper (Th) epitope E7_44–60_ [23–25]. Both DB and PL induced high levels of IFN-γ secreting Th cells with or without EP comparable across the groups (Fig. 1c). The levels of IL-4 secreting Th cells were low across all the experimental groups. Overall there was strong dominance of Th1 responses in the case of both DB and PL with or without EP.

### Induction of protective immunity against the TC-1 tumour

**Fig. 2:**
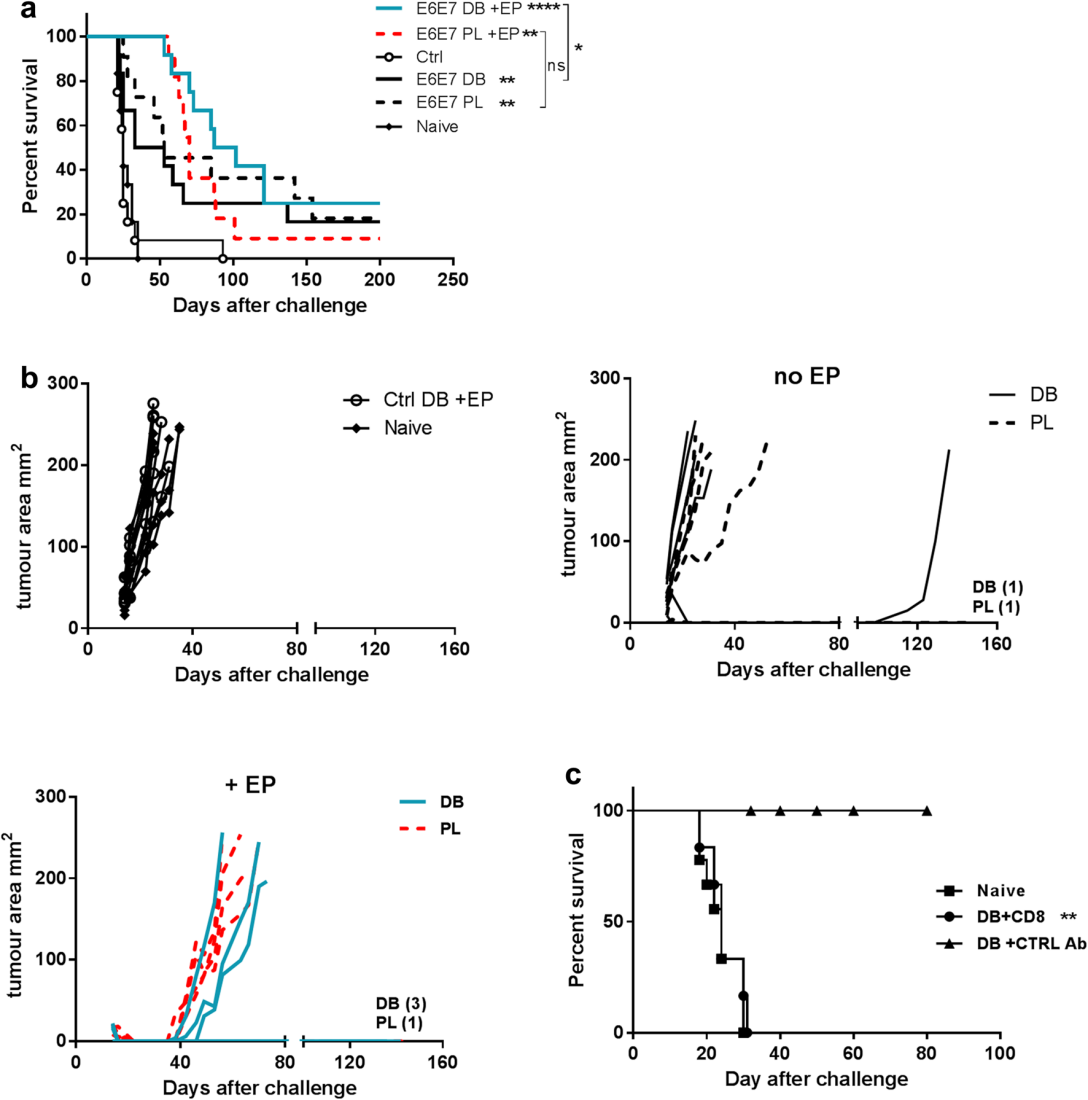
Treatment of TC-1 tumours with DB or PL DNA vaccines encoding E6E7. Mice were injected s.c. with TC-1 tumour cells then on day 3 received either 50 µg of the E6E7 DB or PL vaccines without or with EP, or control DB with EP. Naive mice controls were also challenged with the tumour. **a** Protection of mice is shown. Results combined from two independent experiments, each with *n* = 6 mice per group. **b** The tumour size of individual mice from one representative experiment (of two) is shown. The number of tumour-free mice in each group at the end of the experiments is noted on the graph. **c** An anti-CD8 or control antibody was given to mice vaccinated 4 weeks previously with the DB vaccine plus EP. One day later the mice were challenged with the TC-1 tumour and survival was followed. The antibodies were given again 6 days after tumour challenge. Unvaccinated naïve mice were also challenged with tumour. Combined data from two independent experiments is included. *p < 0.05, **p < 0.01, ****p < 0.0001 compared control DB vaccine + EP unless indicated (a) or DB plus control antibody (c), *ns* non-significant

We next compared the ability of the DB and PL vaccines to confer protection against the TC-1 tumour expressing HPV16 E6 and E7. Mice were injected with the tumour and 3 days later injected with either the DB or PL vaccine with or without EP. Both the tumour size and survival of the mice were monitored (Fig. 2a, b). Overall, variable levels of protection were seen across the groups. Without EP both vaccines performed comparably and conferred a modest but significant protection, however, the majority of mice had only a modest or no delay in the tumour growth. There was a significant delay in tumour growth in mice vaccinated in combination with EP with both vaccines, in comparison to the controls, and some mice never developed the tumour again (Fig. 2a, b).

E6E7 targeting is aimed at induction of CD8 CTLs. Using an anti-CD8 depleting antibody we were able to confirm that the DB vaccine mediated anti-tumour effect was dependent on CD8 T cells (Fig. 2c).

### Induction of antibody against cancer antigens

**Fig. 3:**
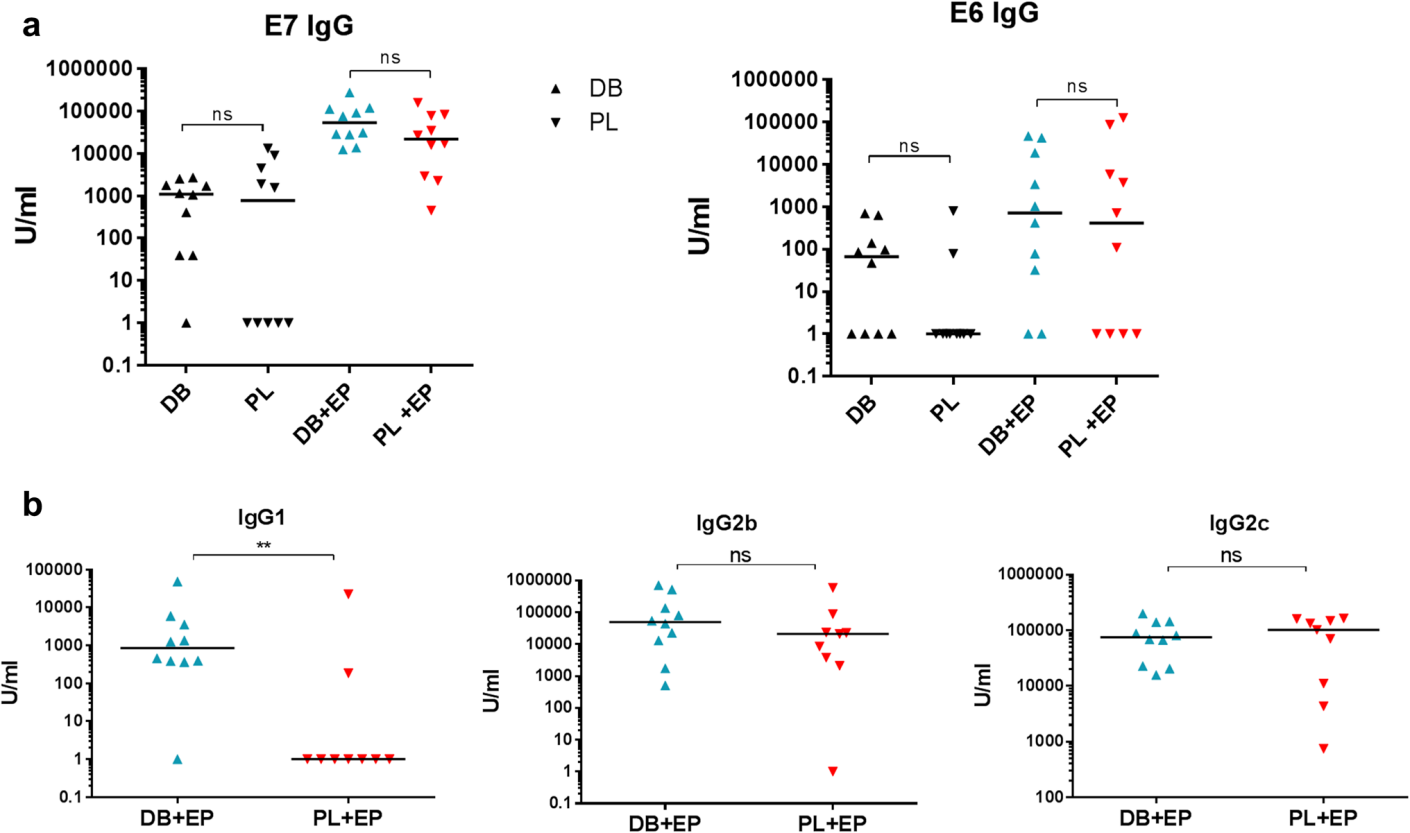
Antibody induction by DB or PL DNA vaccines encoding E6E7. Mice (*n* = 5) were primed and boosted i.m. with 50 µg E6E7 DB or PL vaccines with or without EP and antibody responses measured 2 weeks after the booster injection. **a** Total IgG against E6 or E7. **b** IgG1, IgG2b and IgG2c antibody isotypes measured against E7. Combined data from two independent experiments. **p < 0.01, *ns* non-significant

Although antibody responses are not directly involved in protection against the TC-1 tumour there is scope for antibody to have an effect on antigen presentation via immune complexes and hence induction of CD8 T cells [26]. Additionally for targets located on the cell surface induced antibody can provide an effective way of targeting cancer cells [19, 27]. We therefore, measured total levels of anti-E7 or anti-E6 IgG in individual mice primed and boosted with either the DB or PL vaccines with or without EP. Low levels of IgG were induced to both E6 and E7 proteins without EP (Fig. 3a). Levels were significantly higher with EP and were comparable in both vaccine groups.

The difference between the DB and PL vaccines combined with EP was found in the isotypes of IgG antibody induced: the DB vaccine was able to induce all three isotypes tested while the PL vaccine induced IgG2b and IgG2c but did not induce IgG1 (Fig. 3b). Since the total IgG in the groups without EP was poor the antibody isotypes were not evaluated in those groups.

### Innate immune recognition of DB vaccine

**Fig. 4:**
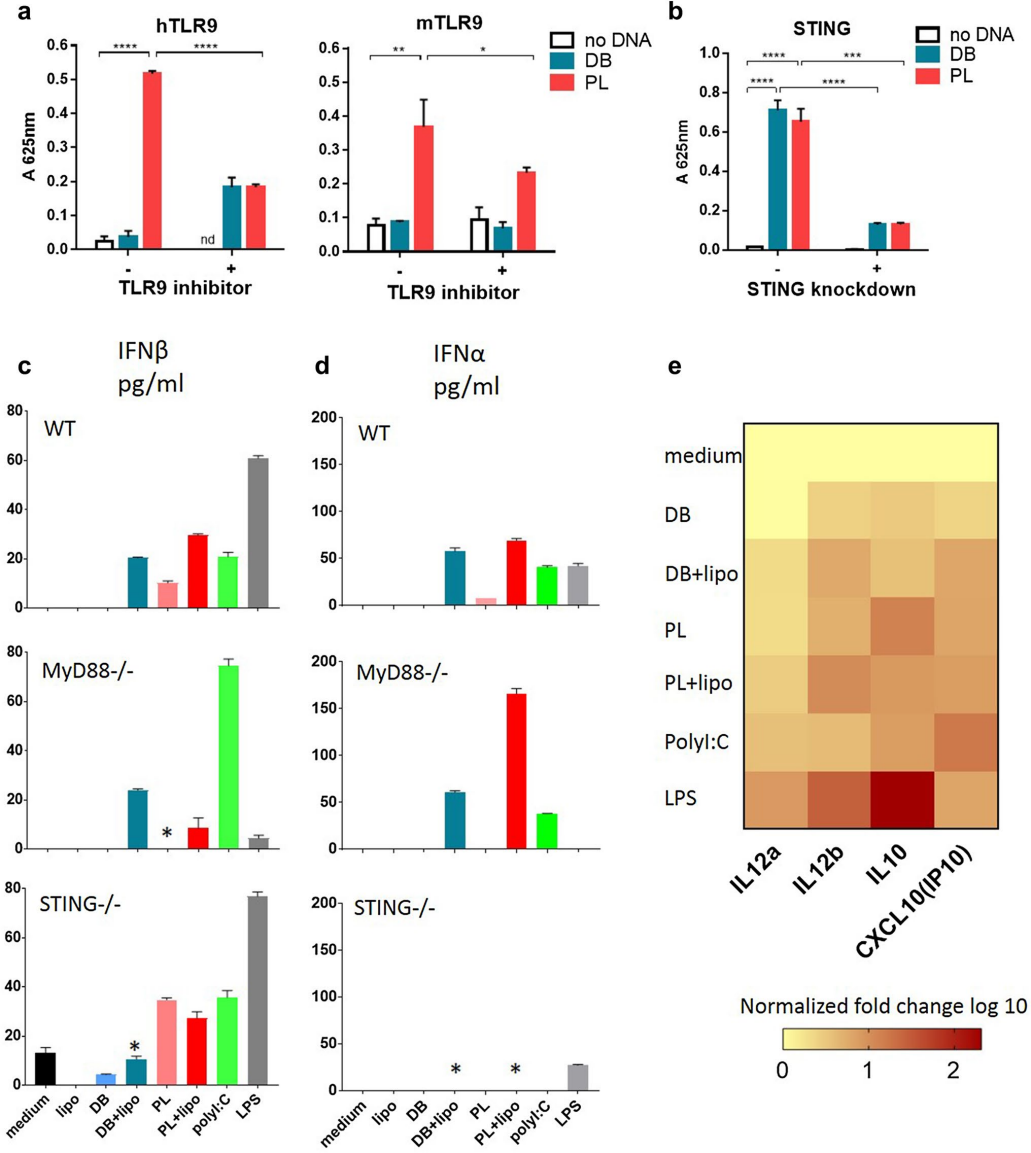
E6E7 dbDNA induces immunity independently of TLR9 stimulation but through cytoplasmic sensors. **a** The E6E7 DB or PL DNA was transfected into the HEK-Blue reporter cell line which expresses human (left panel) or mouse (right panel) TLR9. 10 µg/ml hTLR9 antagonist A151 or mTLR9 antagonist 4084-F was present in specific wells. 42 h after transfection A_625nm_ was measured. Mean is plotted with error bars showing standard deviation. **b** THP1-ISG (STING positive) and THP1 ISG-KD-STING (STING knockdown) reporter cell lines were transfected with E6E7 DB or PL DNA complexed with Fugene, or Fugene alone. Activation of IFN response elements (downstream of STING) was measured by a colour change at A_625nm_. Results show mean change in absorbance of triplicate repeats, compared to blank wells where no transfection took place. Error bars show standard deviation. Results are representative of 3 independent experiments. *p < 0.05, **p < 0.01, ***p < 0.001, ****p < 0.0001. **c, d** Induction of type I interferons following stimulation of BMDC with exogenous or endogenous (+ lipo) DB or PL. **c** ELISA or **d** Luminex in WT, MyD88KO or STINGKO mice. BMDCs were stimulated with DB or PL with or without lipofectamine. After 20 h supernatants were collected. Error bars show standard deviation. The graph is representative of 3 independent experiments. *p < 0.05 when compared to the same treatment in WT. **e** mRNA levels of inflammatory cytokines expressed by BMDCs from WT mice were measured using qPCR. BMDCs were stimulated as in c and d. Levels were normalized to medium control and fold change values are summarised as a heat map

Next, we addressed the question of immune recognition of the DB vaccine by TLR9. HEK-Blue cells expressing either human or mouse TLR9 and the secreted embryonic alkaline phosphatase (SEAP) reporter gene were employed to assay the recognition of DB DNA. When DB DNA was used no effect was observed (Fig. 4a). On the contrary, PL DNA was recognised by both human and mouse TLR9, in keeping with previous findings [3], and the effect could be blocked by a TLR9 specific inhibitor in each case (Fig. 4a). These data indicate that DB DNA is not recognised through TLR9. We next probed recognition of the DB vaccine through intracellular pathways. STING is a key sensor of cytosolic DNA via both direct and indirect mechanisms [28] and we, therefore, used a version of the THP1-Blue IGS reporter cell line with STING knockdown. This cell line otherwise expresses all cytosolic sensors but DNA-dependent activator of IFN-regulatory factors (DAI) [29]. The control THP1-Blue ISG cells expressed functional STING and contain the SEAP reporter gene to measure activation of interferon pathways downstream of STING. Figure 4b shows that DB DNA was recognised through STING regulated cytoplasmic pathways. PL DNA was also able to trigger STING pathways.

To further support the above data, we performed experiments using BMDC generated from WT, MyD88 (adaptor downstream of TLR9) or STING KO mice. DC were treated with either exogenous DB to allow stimulation through TLR9, or lipofectamine transfected DB (endogenous) to stimulate cytosolic sensors [20] and this was paralleled by experiments using PL. The well-defined comparators LPS and poly(I:C) were also included. Type I interferons induced downstream of TLR9 and STING were measured (Fig. 4c, d). Exogenous PL was able to upregulate IFN-β in WT BMDC, while exogenous DB was not as effective (Fig. 4c, d). This ability of PL was reduced in MyD88KO DC, in keeping with TLR9 involvement. Endogenous DB and PL were both effective in WT DC. This ability of DB was reduced in STINGKO demonstrating involvement of the STING pathway in recognition of DB. Of note there was a lesser dependency of PL on STING presumably because endogenous PL also was accessible to TLR9 in the endosomes [30]. For the same reason endogenous PL was affected in MyD88KO while endogenous DB was not. IFN-β was induced only through endogenous delivery of DB or PL and that was reduced in STINGKO mice (Fig. 4d). As reported LPS was able to activate all three types of DC except IFN-β in MyD88KO, while poly(I:C) was effective across the experimental conditions [31].

Th1-related cytokines expression was measured in the supernatants of WT DC treated with DB or PL as above by qPCR (Fig. 4e, Supplementary Fig. 2). Both were able to stimulate IL-12 (IL12a and IL12b encode IL12p35 and IL12p40, respectively), the key regulator of Th1 responses [32]. IP10, reportedly expressed in Th1 responses [33], was induced. IL-10 involved in suppression of IL-12 [32] was also induced. Overall endogenous delivery induced stronger responses.

In conclusion we demonstrated here that the DB DNA vaccine is not recognised by TLR9 but instead recognition of this novel vaccine is through cytoplasmic DNA sensors.

## Discussion

Immunotherapy with monoclonal antibodies targeting checkpoint inhibitors has revolutionised the management of many solid cancers. However, only up to 50% of patients benefit. The challenge is to improve the chances of those who do not, and vaccination is expected to achieve this. The recent success of DNA vaccines with EP in patients with cervical dysplasia may re-open a window of opportunity for DNA vaccines in the clinical cancer setting [11]. The development of a bacteria-free system for production of large amounts of DNA using the DB system aims to meet the growing demand for quality DNA vaccines. Here we explored the ability of this novel type of DNA vaccine to trigger the immune response. This has been addressed by analyzing induction of CD8, CD4 T cells and antibody using a DB DNA vaccine targeting the clinically relevant antigens HPV16 E6 and E7. We demonstrate that this vaccine is able to induce as effective anti-tumour immunity as a PL DNA vaccine, and that the potency of the DB vaccine is improved by EP. Interestingly, even low levels of CD8 T cells induced with one dose of DB without EP were protective. These responses will be likely boosted by the antigenic peptide presented by the tumour cells. The levels of protection induced here is at a similar level previously achieved after a single DNA vaccination [34]. Further improvement could be achieved by boosting. Combination with immunostimulatory/checkpoint antibody is a promising approach to further potentiate DB efficacy as reported for PL [34].

A compelling question has been innate immune recognition of the minimal DNA sequences contained in DB vaccines. PL DNA vaccines are recognised through TLR9 and a number of cytoplasmic sensors [4, 5]. TLR9 recognises unmethylated CpG motifs in the context of XCGY sequences where X is not C and Y is not G. These ISS sequences are more frequently present in bacterial sequences than in mammalian sequences [4]. In the case of DB vaccines immune recognition by TLR9 will depend on the frequency of ISS CpG motifs in the encoded antigen itself since the rest of the vaccine sequence is minimal. Presumably, this will be sufficient in most bacterial antigens. With self-antigens where the frequency of these motifs is low the DB vaccine may be unable to stimulate via TLR9. E6 and E7 oncogenes are not self-antigens but are of a viral origin. Although some viruses have sufficient ISS CpG motifs to stimulate TLR9 [35] the HPV genome has the lowest CpG content among viruses with most of them in non-ISS context, in keeping with HPVs’ association with long-term stable infection [36, 37].

Previously, little dependency on TLR9 for a PL DNA vaccine plus EP has been demonstrated in mice, with the majority of recognition performed through intracellular DNA sensing [38]. It is possible that combination of DNA delivery with EP reduces dependency on TLR9 for immune recognition of DNA vaccines. Although PL DNA was effective at activation of adaptive immunity in mice lacking TLR9, little doubt exists about the involvement of TLR9 in recognition of bacterial DNA in both mice and humans [4, 39]. In mice TLR9 is expressed in both plasmacytoid and conventional DC subsets, while the expression pattern differs in humans and is restricted to plasmacytoid DCs [3, 40, 41]. Here we demonstrated that in contrast to PL DNA, there is a little involvement of either mouse or human TLR9 in recognition of the DB DNA vaccine. On the contrary, DB DNA is able to stimulate intracellular DNA recognition pathways at a similar level to PL DNA. Although no statistically significant differences of tetramer positive CD8 responses were observed between DB and PL, a trend of slightly higher responses was observed in the PL groups possibly accounted for by contribution of TLR9. These findings provide mechanistic insight into the immune recognition of this novel type of DNA vaccine.

The significance of the findings is clear; cytoplasmic recognition of DNA is not restricted to antigen presenting cells; these cytoplasmic DNA sensors are widely expressed as they contribute to the recognition of virally infected cells [42]. The key signalling adaptor for cytosolic DNA sensing, STING, can bind directly to DNA and may participate in direct activation of the STING signalling pathway [42]. Similar to the case for DNA recognition by Absent in Melanoma 2 protein (AIM2) and interferon-gamma-inducible protein 16 (IFI16), cyclic-GMP-AMP (cGAMP) is expressed following stimulation with cytoplasmic DNA can also bind STING [43]. cGAMP is capable of spreading to neighbouring cells which results in stimulation of STING-dependent immune induction in other cells in which DNA is not present in the cytoplasm. These can lead to rapid immune induction without the actual need of DNA spread and can be involved in recognition of DB vaccine.

Interestingly, our in vivo antibody data showed DB with EP also induced IgG1 while PL induced mainly IgG2b and 2c isotypes. This was despite both vaccine groups inducing similar high levels of Th cells secreting IFN-γ, which induces isotype switch to IgG2 isotypes, and similar low levels of Th2 producing IL-4 which drives IgG1 isotype switch. It is likely that in the PL group skewing towards induction of IgG2 isotypes could occur through direct triggering of TLR9 expressed on B cells [44]. Polarization of Th subsets depends on the initial cytokine milieu produced by activated DC. A strong Th1 bias has been demonstrated for DB here similarly to PL with a smaller Th2 component for both. Type I interferons induced by DB and PL through STING, as demonstrated here, are the likely contributors to Th1 polarization [45]. Both DB and PL were able to induce IL-12 the key cytokine for Th1 induction with small amounts of IL-10 which contribute to Th2 responses [32, 46].

Our data show here that in the absence of the ability to stimulate TLR9, the DB vaccine is able to induce a significant adaptive immune response particularly when combined with EP. Taken together, DB which represents a minimal sequence for antigen expression in vivo provides a valuable alternative to PL for applications to patients. Additional sequences including ISS CpG motifs can be easily incorporated into dbDNA™ as desired. The advantage over PL is increased patient safety due to lack of antibiotic resistant genes and endotoxin contamination. These data provide the first preclinical evaluation of DB DNA vaccine targeting cancer antigens.

### Electronic supplementary material

Below is the link to the electronic supplementary material.

Supplementary material 1 (PDF 166 KB)

## Abbreviations

AIM2: Absent in melanoma 2 protein
APC: Allophycocyanin
BMDC: Bone marrow-derived dendritic cells
cGAMP: Cyclic-GMP-AMP
CXCL-10: C-X-C motif chemokine ligand
DAI: DNA-dependent activator of IFN-regulatory factors
DB: Doggybone
dbDNA™: Doggybone DNA
eGFP: Enhanced GFP
EP: Electroporation
FAM: 6-carboxyfluorescein
IFI16: Interferon-gamma-inducible protein 16
IP10: IFN-γ-inducible protein 10
ISS: Immunostimulatory signals
KO: Knock out
PEG: Polyethylene glycol
PL: Plasmid
qPCR: Real-time PCR
SEAP: Secreted embryonic alkaline phosphatase
SFCs: Spot forming cells
STING: STimulator of INterferon Genes
Th: T-helper
