# Supplementary material for: Linear doggybone DNA vaccine induces similar immunological responses to conventional plasmid DNA independently of immune recognition by TLR9 in a pre-clinical model
Source: Cancer Immunol Immunother. 2018 Jan 12;67(4):627–38. doi: 10.1007/s00262-017-2111-y (PMC5860099; doi:10.1007/s00262-017-2111-y)
Supplement: Supplementary file 1 — Supplementary material 1 (PDF 166 KB) [file 262_2017_2111_MOESM1_ESM.pdf]

Cancer Immunology, Immunotherapy (submitted in 2017)- Alex Allen et al.

Supplementary table 1. Overlapping peptide pools for E6 and E7 antigens encoded by DB and PL. Mutations are in red; in bold are peptides corresponding to the linker sequence.

	Peptide no	aa seq
**pool 1**	1	MHGDTPTLHEYMLDL
	2	TPTLHEYMLDLQPET
	3	HEYMLDLQPETTDLY
	4	LDLQPETTDLY**G**Y**G**Q
	5	PETTDLY**G**Y**G**QLNDS
**pool 2**	6	DLY**G**Y**G**QLNDSSEEE
	7	Y**G**QLNDSSEEEDEID
	8	NDSSEEEDEIDGPAG
	9	EEEDEIDGPAGQAEP
	10	EIDGPAGQAEPDRAH
**pool 3**	11	PAGQAEPDRAHYNIV
	12	AEPDRAHYNIVTFCC
	13	RAHYNIVTFCCKCDS
	14	NIVTFCCKCDSTLRL
	15	FCCKCDSTLRLCVQS
**pool 4**	16	CDSTLRLCVQSTHVD
	17	LRLCVQSTHVDIRTL
	18	VQSTHVDIRTLEDLL
	19	HVDIRTLEDLLMGTL
	20	RTLEDLLMGTLGIVC
**pool 5**	21	DLLMGTLGIVCPICS
	22	GTLGIVCPICSQKP**G**
	23	IVCPICSQKP**GGGSS**
	24	ICSQKP**GGGSSGGGS**
	25	KP**GGGSSGGGSG**MFQ
**pool 6**	26	**GSSGGGSG**MFQDPQE
	27	**GGSG**MFQDPQERPRK
	28	MFQDPQERPRKLPQL
	29	PQERPRKLPQLCTEL
	30	PRKLPQLCTELQTTI
**pool 7**	31	PQLCTELQTTIHDII
	32	TELQTTIHDIILECV
	33	TTIHDIILECVYCKQ
	34	DIILECVYCKQQLLR
	35	ECVYCKQQLLRREVY
**pool 8**	36	CKQQLLRREVYDFAR
	37	LLRREVYDFARRDLC
	38	EVYDFARRDLCIVYR

	39	FARRDLCIVYRDGNP
	40	DLCIVYRDGNPYAV**R**
**pool 9**	41	VYRDGNPYAV**R**DKCL
	42	GNPYAV**R**DKCLKFYS
	43	AV**R**DKCLKFYSKISE
	44	KCLKFYSKISEYRHY
	45	FYSKISEYRHYCYSL
**pool 10**	46	ISEYRHYCYSLYGTT
	47	RHYCYSLYGTTLEQQ
	48	YSLYGTTLEQQYNKP
	49	GTTLEQQYNKPLCDL
	50	EQQYNKPLCDLLIRC
**pool 11**	51	NKPLCDLLIRCIN**R**Q
	52	CDLLIRCIN**R**QKPLC
	53	IRCIN**R**QKPLCPEEK
	54	N**R**QKPLCPEEKQRHL
	55	PLCPEEKQRHLDKKQ
	56	EEKQRHLDKKQRFHN
**pool 12**	57	RHLDKKQRFHNIRGR
	58	KKQRFHNIRGRWTGR
	59	FHNIRGRWTGRCMSC
	60	RGRWTGRCMSCCRSS
	61	TGRCMSCCRSSRTRR
	62	MSCCRSSRTRRETQL

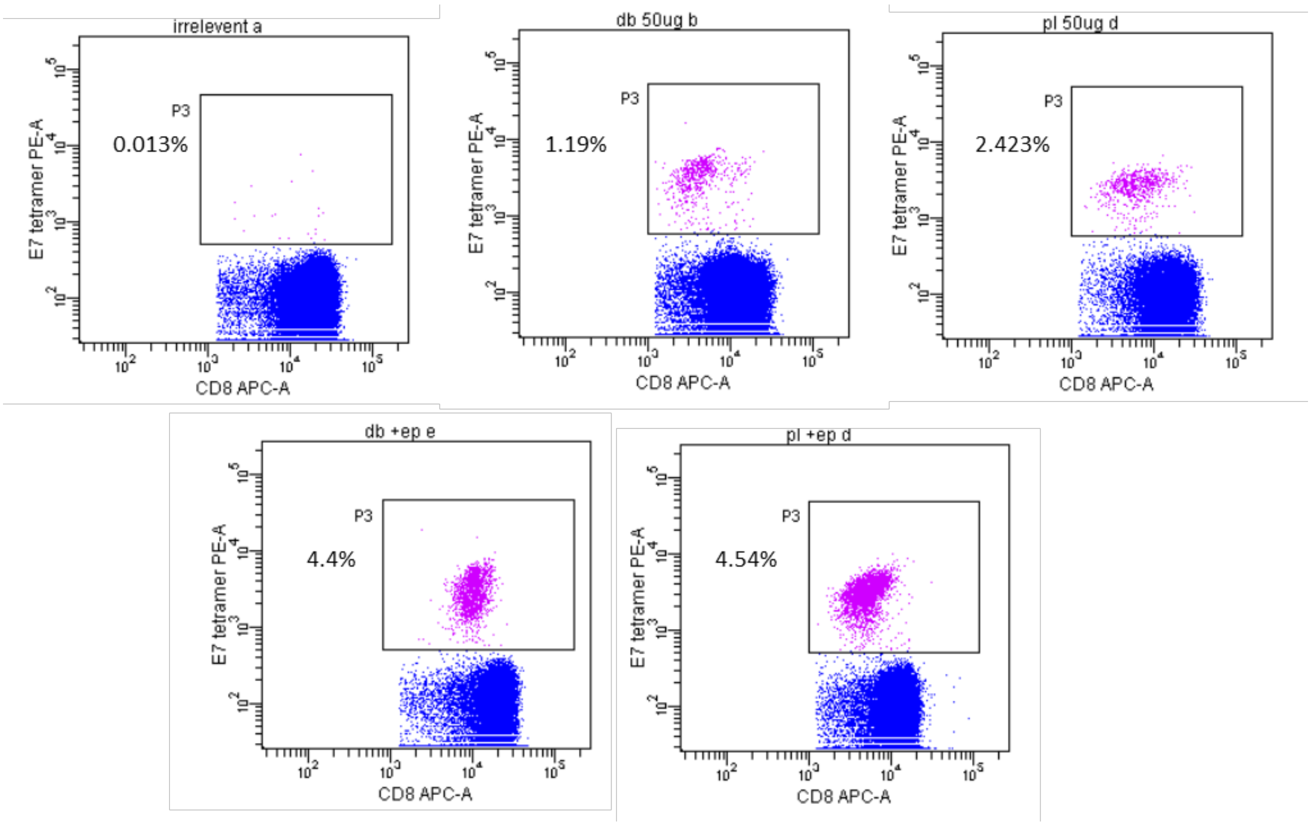

Supplementary figure 1. Representative tetramer staining for DB and PL groups with or without EP alongside of irrelevant DB control. Mice were injected i.m. with 50µg of the E6E7 DB or PL vaccines with or without electroporation and CD8+ responses were measured in blood samples taken after priming on d 14 using PE-labelled H2-Db-$E7_{49\text{-}57}$ tetramer. Values are tetramer positive cells as a percentage of total CD8+ cells.

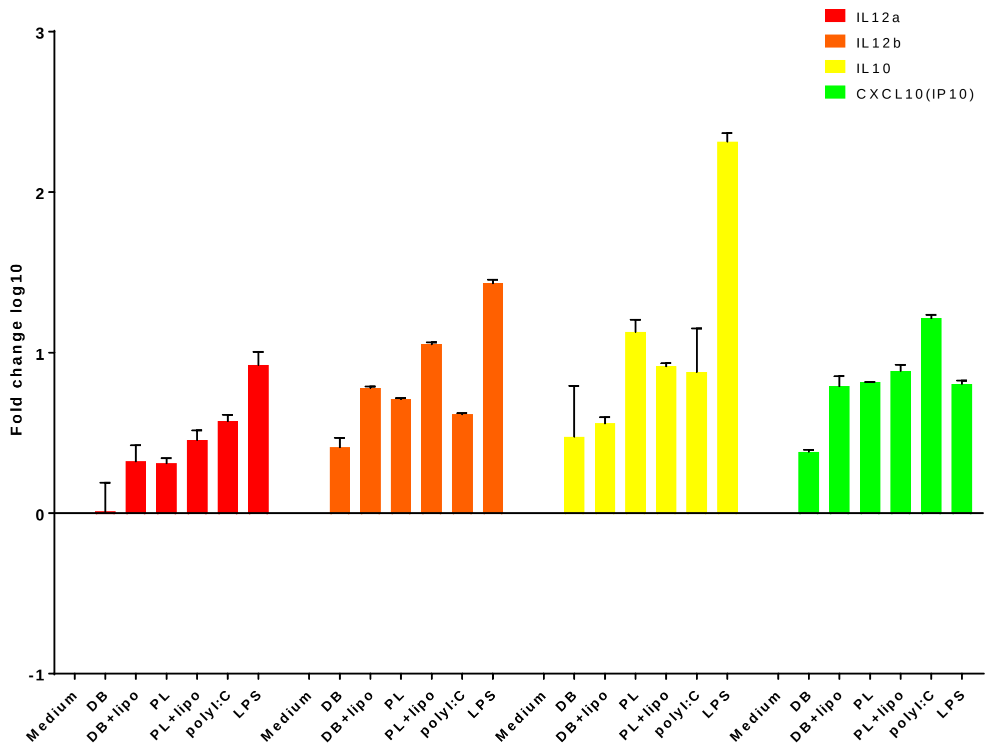

Supplementary figure 2. BMDCs from WT mice were stimulated with 1 μg/ml of DB or PL with 1 μg/ml lipofectamine 2000 (lipo) or without. LPS and poly(I:C) were used as controls at a final concentration of 1 μg/ml. After 20 h following the treatments, cells were collected and mRNA was isolated. mRNA levels of inflammatory cytokines released by BMDCs were measured. The error bars are standard deviations.
